# Nanospheres as the delivery vehicle: novel application of *Toxoplasma gondii* ribosomal protein S2 in PLGA and chitosan nanospheres against acute toxoplasmosis

**DOI:** 10.3389/fimmu.2024.1475280

**Published:** 2024-10-01

**Authors:** WeiYu Qi, YouLi Yu, ChenChen Yang, XiaoJuan Wang, YuChen Jiang, Li Zhang, ZhengQing Yu

**Affiliations:** ^1^ College of animal science and technology, Ningxia University, Yinchuan, Ningxia, China; ^2^ Institute of Animal Science, Ningxia Academy of Agricultural and Forestry Science, Yinchuan, China

**Keywords:** *Toxoplasma gondii*, ribosomal protein S2, nano vaccine, chitosan, PLGA, immune protection

## Abstract

*Toxoplasma gondii* (*T. gondii*) is a zoonotic disease that poses great harm to humans and animals. So far, no effective *T. gondii* vaccine has been developed to provide fully protection against such parasites. Recently, numerous researches have focused on the use of poly-lactic-co-glycolic acid (PLGA) and chitosan (CS) for the vaccines against *T. gondii* infections. In this study, we employed PLGA and CS as the vehicles for *T. gondii* ribosome protein (TgRPS2) delivery. TgRPS2-PLGA and TgRPS2-CS nanospheres were synthesized by double emulsion solvent evaporation and ionic gelation technique as the nano vaccines. Before immunization in animals, the release efficacy and toxicity of the synthesized nanospheres were evaluated *in vitro*. Then, ICR mice were immunized intramuscularly, and immune protections of the synthesized nanospheres were assessed. The results showed that TgRPS2-PLGA and TgRPS2-CS nanospheres could induce higher levels of IgG and cytokines, activate dendritic cells, and promote the expression of histocompatibility complexes. The splenic lymphocyte proliferation and the enhancement in the proportion of CD4^+^ and CD8^+^ T lymphocytes were also observed in immunized animals. In addition, two types of nanospheres could significantly inhabit the replications of *T. gondii* in cardiac muscles and spleen tissues. All these obtained results in this study demonstrated that the TgRPS2 protein delivered by PLGA or CS nanospheres provided satisfactory immunoprotective effects in resisting *T. gondii*, and such formulations illustrated potential as prospective preventive agents for toxoplasmosis.

## 1 Introduction

*Toxoplasma gondii* (*T. gondii*) is a strict intracellular parasite that can infect almost all warm-blooded mammals, including human beings, causing severe zoonotic diseases (1). As estimated, approximately 30% of the world’s population have been infected with *T. gondii* (2). Belonged to the TORCH group of diseases (toxoplasmosis, rubella, cytomegaly, herpes), *T. gondii* can cross the placental barrier, causing teratology, preterm labor, even miscarriage and stillbirth in pregnant women (3). Immunocompetent individuals usually are asymptomatic, while infections in immunocompromised individuals could lead to encephalitis, pneumonia, retinochoroiditis, and other disseminated systemic disease, even death (4, 5). Furthermore, *Toxoplasma* can cause severe economic losses to the livestock industry (6). However, there is currently no effective treatment for toxoplasmosis (7). The most commonly used drugs in clinical practice are sulphonamides and folic acid derivatives, but they have serious side effects (8). Currently, only one vaccine, named Toxovax^®^ (Intervet Inc., Angers, France), was approved by the government of New Zealand for the prevention of abortions in sheep (9). Unfortunately, Toxovax^®^ is only used in the sheep industry and there is a possibility of toxicity recovery (10). Given the limitations of current drugs and the importance of combating toxoplasmosis, there is an urgent need to develop an effective vaccine to prevent the spread of toxoplasmosis.

The prevention of toxoplasmosis urgently requires effective treatment drugs or vaccines, and vaccine prevention is the most effective and cost-effective. Therefore, developing feasible toxoplasmosis vaccines has always been a key research issue. In recent years, different types of vaccines have been developed to prevent toxoplasmosis, including DNA vaccines, live attenuated vaccines, recombinant protein vaccines, and so on (11, 12). However, these vaccines are not effective in preventing toxoplasmosis. The key to vaccine design is mainly the selection of vaccine antigens, and *T. gondii* ribosomal proteins (RPs) are essential in parasite fitness (13). Moreover, *T. gondii* RPs are vital in regulating cell cycle, differentiation, proliferation, and stress response in many organisms (14, 15). *T. gondii* can regulate the transcription of its RP gene when sensing internal pressure in parasites, preventing parasite proliferation (14). In addition, a recent study has demonstrated that the *T. gondii* ribosomal protein S2 (TgRPS2) is localized in the apical end of *T. gondii* tachyzoites and it may be involved in the invasion or egress of *T. gondii* tachyzoites (16). All these studies suggested that TgRPS2 plays an important role in *T. gondii* invasion and survival. Therefore, the TgRPS2 protein, as a promising vaccine antigen, maybe a new direction for vaccine preparation of *T. gondii*.

Natural polymer nanoparticles have received widespread attention in the selection of vaccine delivery because they are easy to prepare and have good biodegradability and biocompatibility (17, 18). Poly-lactic-*co*-glycolic acid (PLGA) is a biodegradable polymers approved by the Food and Drug Administration (FDA) for use in medical products (19). It is widely used in biomedical applications as a drug carrier polymer and can interact with immune system cells to enhance immune efficacy (20, 21). Chitosan (CS) is a naturally occurring polymer approved by the FDA, which has many advantages such as nontoxicity, biocompatibility, and biodegradability (22). Its excellent properties make it widely used in the biomedical field, such as drug carriers, wound dressings, and soft tissue scaffolds, and shows great potential in vaccine delivery (23, 24). PLGA and CS nanospheres as delivery systems for vaccines can prevent premature degradation, improve vaccine antigens stability, and enhance immunogenicity. They enhance immune protection by helping to deliver vaccine antigens to specific locations, inducing innate and adaptive immune responses (17). Recently, PLGA and CS have already been widely used in the delivery of *T. gondii* vaccines and have shown good delivery efficacy (25, 26).

In this study, a novel nano vaccine encoding the TgRPS2 protein was constructed to prevent and control the spread of toxoplasmosis. Recombinant TgRPS2 (rTgRPS2) was obtained through prokaryotic expression, and attempts were made to encapsulate the obtained protein in PLGA and CS nanospheres to develop novel nano vaccines. According to a specific immunization plan, ICR mice were immunized with synthesized TgRPS2-PLGA and TgRPS2-CS nanospheres. Then, the spleens of immunized mice were collected to make paraffin sections, and the antibody levels, cytokine levels, lymphocyte proliferation, and cardiac load of the immunized mice were measured. Finally, the immune protective effect of nano vaccines was assessed based on the above experiments.

## 2 Materials and methods

### 2.1 Parasites, cells, and animals

*T. gondii* RH strain (type I) was gifted by Prof Li Xiangrui from Nanjing Agricultural University and stored at the laboratory. According to previous studies, tachyzoites of the highly pathogenic *T. gondii* RH strain (type I) were intraperitoneally injected into mice for propagation to obtain a purified strain of *T. gondii*. HFF cells were preserved in the research group and maintained in Dulbecco’s Modified Eagle’s Medium (DMEM; Gibco, Carlsbad, CA, USA) containing 10% fetal bovine serum (FBS, Gibco, Carlsbad, CA, USA) and 1% double antibiotics (penicillin-streptomycin solution, New Cell & Molecular Biotech, Suzhou, China) at 37°C in a 5% atmosphere, were used for *T. gondii* generation.

Specific pathogen-free (SPF) ICR mice (18-22 g, female, approximately 7-8 weeks) and Wistar rats (200-220g, female, approximately 6-8 weeks) were obtained from Beijing Vital River Laboratory Animal Technology Co., Ltd and kept in a strict SPF environment. ICR mice are internationally recognized as closed-group mice, and widely used in safety evaluation, pharmacology, toxicology, and immunology research. This study was approved by the Ningxia University Technology Ethics Committee (No: NXU-2023-085, No: NXU-2023-086). Under the supervision of the Ningxia University Technology Ethics Committee, all relevant animal operations were performed in strict compliance with the Chinese legislation regarding the use and care of research animals (GB/T35823-2018).

### 2.2 Cloning and expression of recombinant TgRPS2

The FreeZol Reagent (Vazyme Biotech Co., Ltd, Nanjing, China) was used to extract total RNA from 10^7^ *T. gondii* tachyzoites based on the instructions provided. cDNA preparation was completed according to the reverse transcription kit protocol (ABclonal, Wuhan, China). To amplify the complete open reading frame (ORF) of TgRPS2, a pair of specific primers were designed based on the conserved domain sequences (CDS) of TgRPS2 (GenBank: TGRH88_017180): forward primer, 5’-CCGGAATTCATGGCAGAACGCGGCAGC-3’; reverse primer, 5’-CCCAAGCTTCTAGGCGAGCGGTCGCGAC-3’ (The underlined sequences respectively represent the restriction endonuclease of *EcoR*I and *Hind*III), were synthesized by Sangon Biotech (Xian, China). Following the instructions of 2 × Phanta Max Master Mix (Vazyme Biotech Co., Ltd, Nanjing, China), CDS fragments of TgRPS2 were obtained by PCR amplification. Subsequently, the appropriate bands were identified by 1.5% agarose gel electrophoresis containing 0.01% ethidium bromide, and the correct bands were purified by Gel Extraction Kit (Vazyme Biotech Co., Ltd, Nanjing, China). Then, purified PCR products and the pET32a vector (Takara Biotechnology, Dalian, China) were digested by restriction endonucleases *EcoR*I and *Hind*III (Takara Biotechnology, Dalian, China) and ligated using T4 DNA ligase (Takara Biotechnology, Dalian, China) to obtain the pET32a-RPS2 recombinant plasmid. The constructed plasmids were validated by double restriction enzyme digestion and then sequenced on an ABI PRISM™ 3730 XL DNA Analyzer (Applied Biosystems, Waltham, MA, USA) by Sangon Biotech (Xian, China).

After obtaining the correct recombinant plasmid pET32a-RPS2, it was transformed into *Escherichia coli* BL21 (DE3) (Tsingke Biological Technology, Nanjing, China) and then amplified and cultured in Luria Bertani medium (LB) (37°C with shaking at 180 rpm). Subsequently, it was expressed under the induction of 1 mM isopropyl-β-d-thiogalactopyranoside (IPTG). Analysis of recombinant protein expression by 12.5% sodium salt polyacrylamide gel electrophoresis (SDS-PAGE) and stained with Coomassie blue. Then, based on the distribution of proteins, they were filtered with 0.22 μm membrane (BioSharp, Hefei, China) and purified by a chelating nickel column (Cytiva, Logan, UT, USA). Purified proteins were concentrated by the addition of polyethylene glycol (PEG) powder and the purification effect was confirmed by SDS-PAGE. The endotoxin was removed from the purified protein following the instructions provided with the ToxinEraser™ Endotoxin Removal Kit (GeneScript, Piscataway, NJ, USA). Subsequently, the ToxinSensor™ Chromogenic LAL Endotoxin Assay Kit (GeneScript, Piscataway, NJ, USA) was employed to assess the endotoxin levels in the recombinant proteins before further analysis. The recombinant protein concentration was determined by the Bicin-choninic Acid (BCA) protein quantification kit (New Cell & Molecular Biotech, Suzhou, China) and stored at -80°C.

### 2.3 Preparation of anti-rTgRPS2 polyclonal antibody

The polyclonal anti-rTgRPS2 antibody was produced in Wistar rats. Before immunization, blank sera were collected from rat eye sockets as a control. Then, rats were first immunized subcutaneously at four different sites with 300 μg of purified rTgRPS2 protein emulsified with an equal volume of Freund’s complete adjuvant (Sigma-Aldrich, St. Louis, MO, USA). Two weeks later, rats were immunized with the same dose of purified rTgRPS2 protein emulsified with Freund’s incomplete adjuvant (Sigma-Aldrich, St. Louis, MO, USA) at one-week intervals. A total of eight immunizations were administered, and blood containing polyclonal antibodies was collected from the rat eye sockets every 7 days after the sixth immunization. All sera were stored at -20°C until use.

### 2.4 Isolation and purification of soluble tachyzoite antigens from *Toxoplasma gondii* and generation of anti-*T. gondii* STAg polyclonal antibody

In order to obtain soluble tachyzoite antigens (STAg) of *T. gondii*, 5×10^6^ tachyzoites were resuspended in 1 ml of phosphate-buffered saline (PBS). Subsequently, protease and phosphatase inhibitors (Beyotime, Shanghai, China) were added, and the tip sonication (Scientz Biotechnology, Ningbo, China) was carried out in continuous mode for 2 s (15 min in total) at 30 W output power. The obtained proteins were quantified using the BCA protein quantification kit (Beyotime, Shanghai, China), and were stored at -20°C until use. Similarly, we obtained polyclonal antibodies against *T. gondii* STAg using the same immunization strategy as in 2.3.

### 2.5 Western blotting analysis

To analyze the reactogenicity between the natural TgRPS2 proteins and the polyclonal antibodies against the recombinant TgRPS2 proteins, the purified rTgRPS2 protein or *T. gondii* STAg were first separated by 12.5% SDS-PAGE gel, and then transferred to a polyvinylidene difluoride (PVDF) membrane (Millipore, Billerica, MA, USA). Subsequently, TBST (TBS containing 0.5% Tween 20) containing 5% (*w/v*) skim milk powder was used to block non-specific binding sites at 37°C for 1.5 hours. After that, being washed in TBST for three times, the membranes were incubated with sera against STAg (1:100 dilution) or sera against rTgRPS2 (1:100 dilution) overnight at 4 °C. After being rinsed in TBST for five minutes, the membranes were stained with HRP-conjugated goat anti-Rat IgG (1:8000 dilution, ABclonal, Wuhan, China) at 37°C for 1 hour. Rinsed again in TBST for five minutes, the membranes were finally imaged by Tanon™ Femto-sig ECL Western Blotting Substrate (Tanon, Shanghai, China) under a chemiluminescent image analysis system (Tanon, Shanghai, China).

### 2.6 Preparation of nanospheres

PLGA nanospheres were synthesized using the double emulsion solvent evaporation technique (*w*/*o*/*w*) (27, 28). Briefly, 0.6 g of polyvinyl alcohol (PVA, MW 31,000–75,000 Da, Macklin, Shanghai, China) was weighed and dissolved in an appropriate amount of deionized water. Then a container was placed on a magnetic stirrer stirring at 500 rpm until the PVA was completely dissolved. Adjust the volume to 20 ml to prepare a 3% PVA solution. Subsequently, 100 mg of PLGA was mixed with 1 ml of dichloromethane (DCM, Sigma, Saint Louis, MO, USA) into a 50 ml centrifuge tube to form the organic phase. At room temperature, 2 ml of 3% PVA solution was stirred using a magnetic stirrer, and 2 ml of rTgRPS2 protein (at a concentration of 1 mg/ml) was dripped with stirring. After mixing, tip sonication was carried out at 4°C for 6 min in continuous mode at an output power of 35 W for 2 s at a time to synthesize *w/o* emulsion by Tip sonication (Scientz Biotechnology, Ningbo, China). Then 3 ml of 3% PVA solution was dropped into the *w/o* emulsion, which was mixed by a magnetic stirrer and then subjected to tip sonication to synthesize the *w/o/w* emulsion. Subsequently, the obtained *w/o/w* emulsion was transferred to a 50 ml centrifuge tube for overnight evaporation of DCM. Then, centrifuge at 40,000 r/min at 4°C for 20 minutes to separate the supernatant from the precipitate. Resuspend the precipitate in deionized water and adjust the volume to 4 ml. Transfer the resuspension to a lyophilization vial, freeze it at -80°C for at least 2 hours, and then transfer it to a vacuum freeze-drier (Yiheng, Shanghai, China) for 24 hours until completely freeze-dried. The freeze-dried PLGA nanospheres were stored at 4°C under sealed conditions until use.

The desired CS nanospheres were synthesized using the ionic gelation technique with slight modifications (29). Briefly, 100 mg of CS (MW 50-190 kDa, Macklin, Shanghai, China) was dissolved in 50 ml of 1.0% (*v/v*) aqueous acetic acid solution on a magnetic stirrer (400 rpm) until complete dissolution. Subsequently, the solution pH was adjusted to 4.6-5 by adding appropriate 20 wt% aqueous sodium hydroxide solution. 40 mg of TPP was dissolved in 20 ml of double-distilled water, and the completed 2 mg/ml sodium tripolyphosphate (TPP, Aladdin, Shanghai, China) solution was prepared by passing it through a 0.22 μm filter membrane (BioSharp, Hefei, China). 10 ml of CS solution was transferred to a 50 ml centrifuge tube, and then 2 mg of rTgRPS2 protein (at a concentration of 1 mg/ml) was cautiously dripped to 2 ml of TPP solution for 20 min on a magnetic stirrer stirring at 500 rpm. Then, the tip sonication was carried out for 5s at 2 s intervals (5 min total) under output power of 45 W. After sonication, the samples were centrifuged at 40,000 rpm for 20 min at 4°C, and the obtained nanospheres were rinsed and resuspended in double-distilled water and passed through a 0.22 μm filter membrane. After freezing at -80°C, the CS nanospheres were transferred to a vacuum freeze dryer until completely freeze-dried, and then the freeze-dried CS nanospheres were stored at 4°C under sealed conditions until use.

### 2.7 Loading capacity and encapsulating efficacy of the nanospheres

The morphological characteristics of synthesized nanospheres were analyzed using scanning electron microscopy (SEM) from the College of Life Science, Nanjing Agriculture University, PR China, and the average diameter of the PLGA and CS nanospheres was quantified using ImageJ software (version 1.8, NIH Image, Bethesda, MD, USA).

To determine the loading capacity (LC) and encapsulation efficiency (EE) of the synthesized nanospheres, the concentration of non-bound proteins in the supernatants of the collected PLGA and CS nanospheres was determined using the BCA protein quantification kit (New Cell & Molecular Biotech, Suzhou, China). The LC and EE of PLGA and CS nanospheres were calculated according to (Equations 1, 2), respectively.

To investigate the release characteristics of TgRPS2-PLGA and TgRPS2-CS nanospheres *in vitro*, two nanospheres were dispersed in PBS (pH 7.4) under magnetic stirring at 180 rpm at 37°C. Every 12 h, the supernatant samples were collected by centrifugation and stored at -20°C. After each collection, the settled nano drops were resuspended with the original solution. After the last collection, the protein concentration in the sample was measured using the BCA protein quantification kit (New Cell & Molecular Biotech, Suzhou, China), and the cumulative release (CR) was calculated using the (Equation 3).


(1) $$LC\left(\%\right)=\frac{Total\;protein-Free\;protein\;concentration\times volume\;of\;supernatant}{weight\;of\;nanospheres}\times 100\%$$

(2) $$EE\left(\%\right)=\frac{Total\;protein-Free\;protein\;concentration\times volume\;of\;supernatant}{Total\;protein}\times 100\%$$

(3) $$CR\left(\%\right)=\frac{protein\;concentration\times volume\;of\;supernatant}{Total\;protein}\times 100\%$$

### 2.8 Nanosphere toxicity assessment

A total of 35 ICR mice were randomly allocated to seven experimental groups (n = 5 mice): Blank (immunized with an equal volume of PBS), Control (immunized with an equal volume of the pET32a-label protein), PLGA (immunized with an equal volume of PLGA nanospheres), CS (immunized with an equal volume of CS nanospheres), TgRPS2 (immunized with an equal volume of TgRPS2 protein), TgRPS2-PLGA (immunized with an equal volume of TgRPS2-PLGA nanospheres), and TgRPS2-CS (immunized with an equal volume of TgRPS2-CS nanospheres). Mice were immunized with a triple dose of the regular dosage, and 300 μg of protein was injected intramuscularly in each mouse. Three days later, a booster immunization was administered using the same strategy. One day after the completion of the immunization procedure, sera were collected from the eye sockets of the mice. The serum levels of creatinine (Cr) and blood urea nitrogen (BUN) were assessed using the sarcosine oxidase assay (Solarbio, Beijing, China) and the urease-indole phenol assay (Solarbio, Beijing, China). During the trials, the physical health and mental state of the mice were observed and recorded daily.

### 2.9 Immunization schedule and *T. gondii* challenge

ICR mice were randomly divided into 7 groups (n=26). Before immunization, all TgRPS2 proteins and synthesized nanospheres were diluted and suspended in PBS (pH 7.4). Then mice were immunized by multipoint intramuscular injections according to the immunization schedule (**Table 1**). Briefly, on the 1 and 15 days, mice were given their first and booster immunizations according to their groups. Simultaneously, blood and splenic lymphocytes were collected from the mice after 10 days of each immunization, and serum samples were preserved at -20°C. After 14 days of the booster immunization (on the 29th day), all mice were challenged with 100 tachyzoites from highly virulent *T. gondii* RH strains intraperitoneally as indicated previously (30). Four days after the challenge experiment (on the 33th day), three mice were anesthetized and sacrificed under the supervision of the Ningxia University Technology Ethics Committee, and spleen tissues were isolated in 10% neutral buffered formalin. Six days after the challenge (on the 35th day), ten mice were anesthetized and sacrificed under supervision, and cardiac muscles were collected and stored at -20°C.

**Table 1:** Group assignment and immune procedure.

Group	Immunization strategy (first immunization and booster immunization on day 1 and day 15)	Challenge strategy (14 days after the booster immunization)
Blank	An equal volume of PBS	Intraperitoneal injection of 100 highly virulent RH strains of *T. gondii* on day 29
Control	100 μg of the pET32a label protein	
PLGA	100 µg of blank PLGA nanospheres	
CS	100 µg of blank CS nanospheres	
TgRPS2	100 µg of TgRPS2 protein	
TgRPS2-PLGA	TgRPS2-PLGA nanospheres containing 100 μg of TgRPS2 protein	
TgRPS2-CS	TgRPS2-CS nanospheres containing 100 μg of TgRPS2 protein	

### 2.10 Antibody and cytokine assays in immunized mice

The titers of *T. gondii*-specific antibodies in mice serum were determined using an indirect enzyme-linked immunosorbent assay (ELISA). Briefly, each well in the ELISA plate was coated with 1 μg of STAg (diluted to 10 μg/ml with carbonate buffer pH 9.6) overnight at 4°C. After rinsing in TBST 5 times, each well was blocked with TBST containing 5% bovine serum albumin (BSA) at 37°C for 1 h (Sangon Biotech, Shanghai, China).

The plates were washed three times with TBST. The wells were then incubated with sera (diluted 1:100 in TBST containing 5% BSA) from immunized mice at 30°C for 1 h. After being washed with TBST three times, the plates were incubated with HRP-conjugated anti-mouse IgG, IgG1, or IgG2a (1:5000, ABclonal, Wuhan, China) at 37°C for 1 h. The plates were washed again, and the enzymatic activity was assessed using 3,3′,5,5′-tetramethylbenzidine (TMB, Tiangen, Beijing, China) as the substrate. The reaction was conducted in the dark at room temperature for 10 minutes. Finally, the reaction was stopped with 100 μl of freshly prepared H_2_SO_4_. A microplate photometer (BioTek, Burlington, VT, USA) was used to quantify the titers of antibodies at an absorbance of 450 nm.

The secretion of cytokines interferon-γ (IFN-γ), transforming growth factor β (TGF-β), IL-4, IL-6, IL-10, and IL-17 in mice serum was determined using a commercial ELISA kit (Enzyme Linked Biotechnology Co., Ltd, Shanghai, China) according to the instructions.

### 2.11 Flow cytometry analysis

After 10 days of the first and the booster immunization, respectively, five mice from each group were sacrificed before immunization or challenge, and splenic lymphocytes were collected using a lymphocyte isolation kit (Solarbio, Beijing, China). The percentages of CD4^+^ and CD8^+^ T cells of mice were detected using flow cytometry. Isolated spleen lymphocytes were cultured overnight in DMEM containing 10% FBS and 1% double antibiotics. Non-adherent cells were discarded, and adherent cells were collected after washed with PBS three times. Then, cells were adjusted to 10^6^ cells in 100 μl PBS. The spleen lymphocytes were, respectively, stained with anti-mouse CD3e-PE and CD4-FITC (eBioscience, San Diego, CA, USA), and anti-mouse CD3e-PE and CD8-FITC (eBioscience, San Diego, CA, USA) in the dark for 40 minutes at 4°C. In order to investigate the changes of CD83, CD86, and MHC molecules in splenic DCs, isolated lymphocytes were double-stained with anti-mouse CD11c-APC, CD86-FITC, CD83-PE, and anti-mouse CD11c-PE, MHC-I-FITC, and MHC-II-APC antibodies (eBioscience, San Diego, CA, USA) in the dark for 40 minutes at 4°C. After being washed three times in PBS, centrifugation, and collection, cells were sorted using flow cytometry (Beckman Coulter Inc., Brea, CA, USA).

### 2.12 Lymphocyte proliferation assay

The day before the challenge experiment (On the 28th day), three mice from each group were sacrificed, and spleen lymphocytes were isolated using the same method as described in 2.11. Splenic lymphocytes were seeded into a 96-well plate at a density of 5 × 10^5^ cells per well. The cells in each well were stimulated with rTgRPS2 (10 μg/ml) and incubated for 48 h at 37°C in a 5% CO_2_ incubator. Afterward, Cell Counting Kit 8 (CCK-8, Beyotime, Shanghai, China) was added to each well according to the manufacturer’s instructions and incubated for 4 h. Subsequently, the absorbance at 450 nm was measured using a microplate reader (BioTek, Burlington, VT, USA) to assess lymphocyte proliferation.

### 2.13 Immunohistochemical analysis of the spleen from immunized mice

After 14 days of the booster immunization (on the 29th day), all mice were challenged with 100 tachyzoites from highly virulent *T. gondii* RH strains intraperitoneally. Four days after the challenge, three mice were sacrificed under the supervision, and spleen tissues were isolated from mice, and fixed in 10% neutral buffered formalin for 24-48 hours. Subsequently, dehydrate the tissue gradually in increasing ethanol concentrations for 1-2 hours each, followed by clearing in xylene for 30 minutes. Embed the spleen tissue in paraffin and sectioned at a 6 μm thickness using Minux^®^ Rotary Microtomes S710 (RWD Life Science, Shenzhen, China). The sections were dehydrated and then dried for 24 h at 37°C, and inactivated with 3% H_2_O_2_ for 25 min under dark conditions to inhibit endogenous peroxidase activity. Subsequently, the sections were blocked with blocking solution (5% bovine serum albumin) at 37°C for 30 min and incubated with anti-*T. gondii* antibody (1:500 dilution, Abcam, Cambridge, UK) at 4°C overnight. After being washed with PBS three times, the sections were incubated with HRP-conjugated goat anti-rabbit IgG (1:8000 dilution, ABclonal, Wuhan, China) at room temperature for 1 h. Finally, the sections were detected by prepared 3,3′-diaminobenzidine (DAB; Sangon Biotech, Shanghai, China) for 5 min and were counterstained with hematoxylin, then dehydrated and sealed with neutral balsam. The sections were examined under a microscope to assess the distribution of *T. gondii* in the spleen tissues.

### 2.14 Parasite burden in mice

To analyze the immune protective efficacy elicited by nano vaccines, absolute quantitative real-time PCR (qPCR) was performed to investigate *T. gondii* burdens in the cardiac tissue of challenged animals. Six days after the challenge, ten mice were sacrificed under the supervision, and the cardiac tissue was collected. Then, genomic DNA was extracted from 30 mg of cardiac tissue collected from the challenged animals following the manufacturer’s instructions for the genomic DNA extraction kit (OMEGA Bio-tek, Norcross, GA, USA). The purity of DNA samples was evaluated by measuring the OD260/OD280 ratio using a nanodrop microvolume spectrophotometer (Thermo Scientific, Waltham, MA, USA). The recombinant vector was constructed according to previous studies (31), and the copy number was calculated. The 529 bp fragments in the extracts were amplified by ChamQ Universal SYBR qPCR MasterMix (Vazyme Biotech Co., Ltd, Nanjing, China) using a CFX96 amplifier (Bio-rad, Hercules, CA, USA). Before further analysis, the melting curve of each amplification was verified, and each displayed a single uniform peak as expected.

### 2.15 Statistical analysis

One-way ANOVA with multiple comparisons was conducted using GraphPad Prism 9.5 software (GraphPad Prism, San Diego, CA, USA) to account for differences between groups. Statistical significance was determined at *p* < 0.05, and the results are presented as mean ± SD for each group. Flow cytometry analysis was conducted using CytExpert software (version 2.3, Beckman Coulter Inc., Brea, CA, USA).

## 3 Results

### 3.1 Plasmid construction

**Figure 1:**
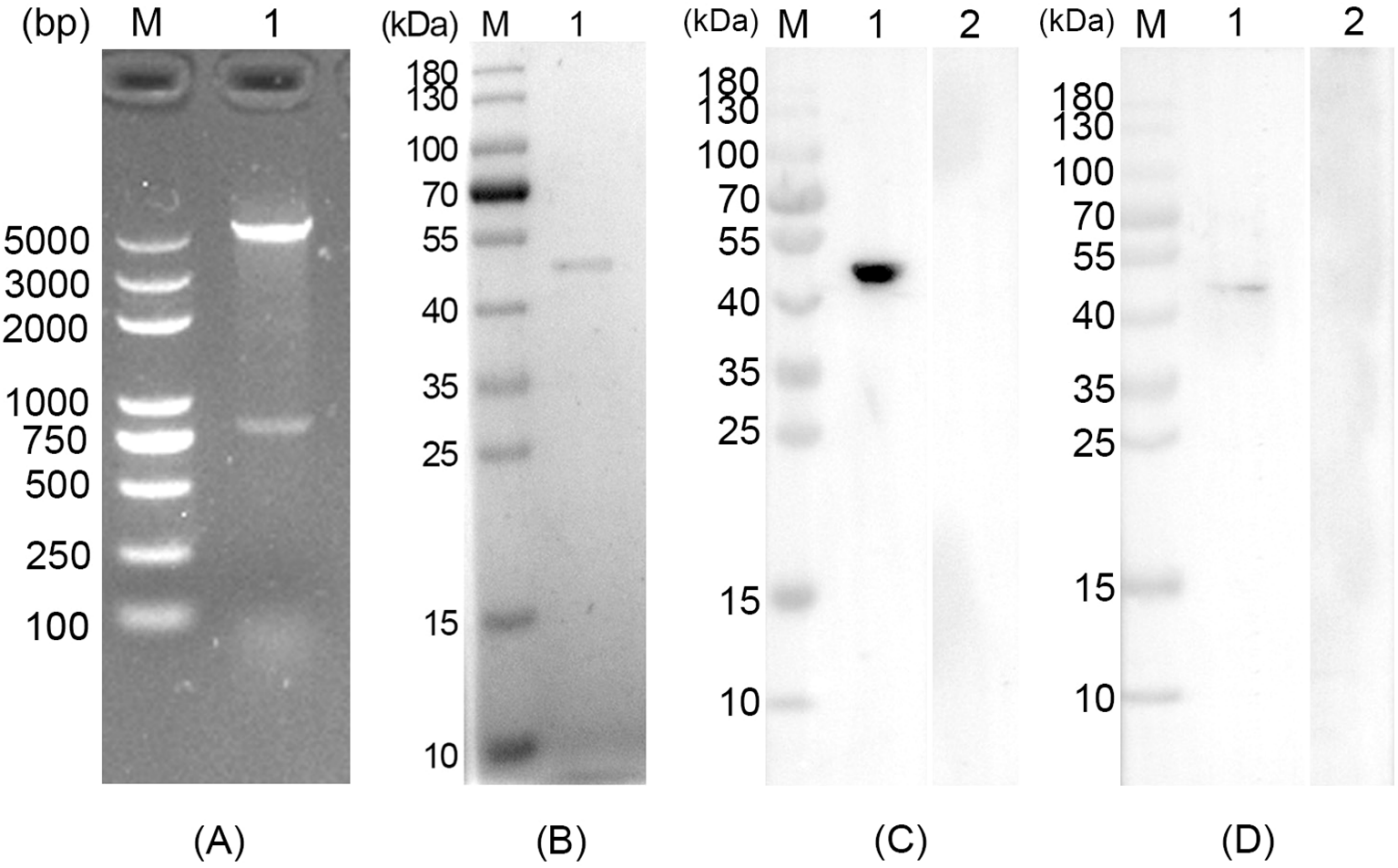
**(A)** Double digestion analysis of the recombinant plasmid pET32a-RPS2. Line M: DL5000 marker, Line 1: Double digestion with *EcoR*I and *Hind*III. **(B)** SDS-PAGE analysis of the purified recombinant *Toxoplasma gondii* ribosomal protein RPS2 (rTgRPS2). Line M: pre-stained protein marker (10-180 kDa), Line 1: Purified rTgRPS2 protein. **(C)** Anti-*T. gondii* STAg antibodies recognized rTgRPS2 proteins. Line M: pre-stained protein marker (10-180 kDa), Line 1: anti- *T. gondii* antibody recognized rTgRPS2, Line 2: rat negative serum recognized rTgRPS2. **(D)** Anti-rTgRPS2 protein serum recognized *T. gondii* STAg. Line M: pre-stained protein marker (10-180 kDa), Line 1: anti-rTgRPS2 rat serum recognized *T. gondii* STAg, Line 2: rat negative serum recognized *T. gondii* STAg.

The obtained pET32a-RPS2 plasmid was first double-digested by *Eco*RI and *Hin*dIII, and then verified in 1% agarose gel electrophoresis, respectively generating two fragments of 816 bp and 5881 bp (**Figure 1A**). In accordance with the expected result, the obtained results revealed that the inserted fragment was the correct ORF of TgRPS2. In addition, DNA sequencing showed that the target fragments were correctly inserted in the pET32a vectors. In conclusion, all the results mentioned above indicated that the pET32a-RPS2 plasmid was constructed correctly.

### 3.2 Expression, purification, and western blot analysis of recombinant TgRPS2

The molecular weight of recombinant TgRPS2 was 47.3 kDa, which included pET32a vector protein (18.0 kDa) and native TgRPS2 protein (29.3 kDa). As shown in **Figure 1B**, the results of SDS-PAGE showed that the molecular weight of the purified rTgRPS2 protein was about 47 kDa, which conformed to the expected result. Furthermore, western blot analysis revealed that anti-*T. gondii* STAg antibodies could react with the rTgRPS2 protein (**Figure 1C**), emphasizing that the rTgRPS2 protein could be recognized by anti-*T. gondii* STAg antibodies. The interaction of STAg from *T. gondii* with serum containing anti-rTgRPS2 antibodies demonstrated that the anti-rTgRPS2 antibodies could effectively recognize the STAg from *T. gondii* (**Figure 1D**). All these results suggest that rTgRPS2 exhibits good immunogenicity, which can be used as the vaccine antigen.

### 3.3 Morphology of nanospheres

**Figure 2:**
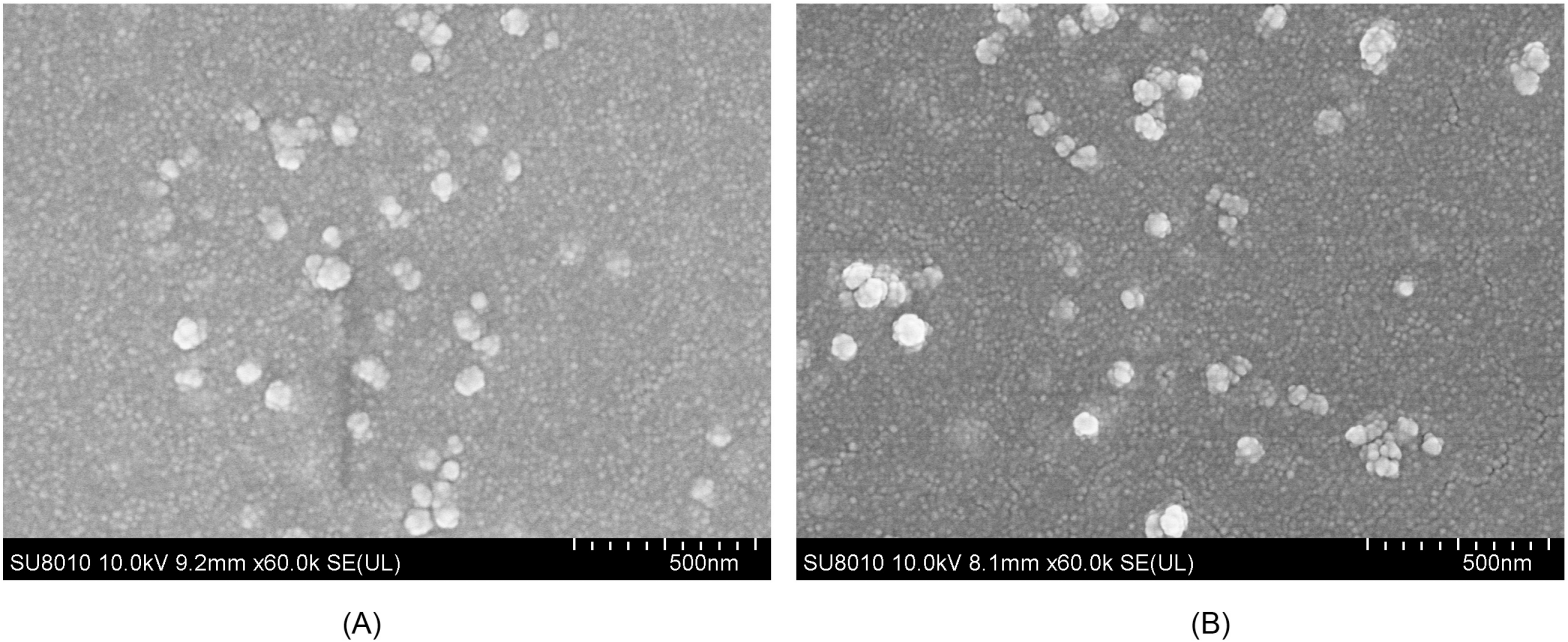
SEM images of TgRPS2 protein entrapped in PLGA **(A)** and CS nanospheres **(B)**. By double emulsion solvent evaporation (*w/o/w*) and ionic gelation technique, TgRPS2-PLGA and TgRPS2-CS nanospheres were synthesized. After being completely freeze-dried, nanospheres were imagined at a magnification of ×30,000 (bar represented 500 nm) by an SEM.

After encapsulation of the rTgRPS2 protein in PLGA and CS nanospheres, the morphology of the synthesized nanospheres was observed using SEM. As illustrated in **Figure 2**, the nanospheres appeared spherical with uneven surface textures, and slight irregularities were also detected. Employed by Image J software, the diameters were analyzed according to five randomly selected nanospheres. The average diameter of TgRPS2-PLGA and TgRPS2-CS nanospheres were 79.46 ± 6.14 nm and 93.98 ± 14.37 nm by analyzing the SEM pictures (n = 5). By conducting the concentration of nonbinding proteins and total proteins in the supernatant using BCA protein quantification kit, the LC values of PLGA and CS nanospheres were 0.94% and 5.15% (n = 3) respectively, while EE values were 70.33% and 41.45% (n = 3).

### 3.4 Slow-release profile of nanospheres *in vitro*

**Figure 3:**
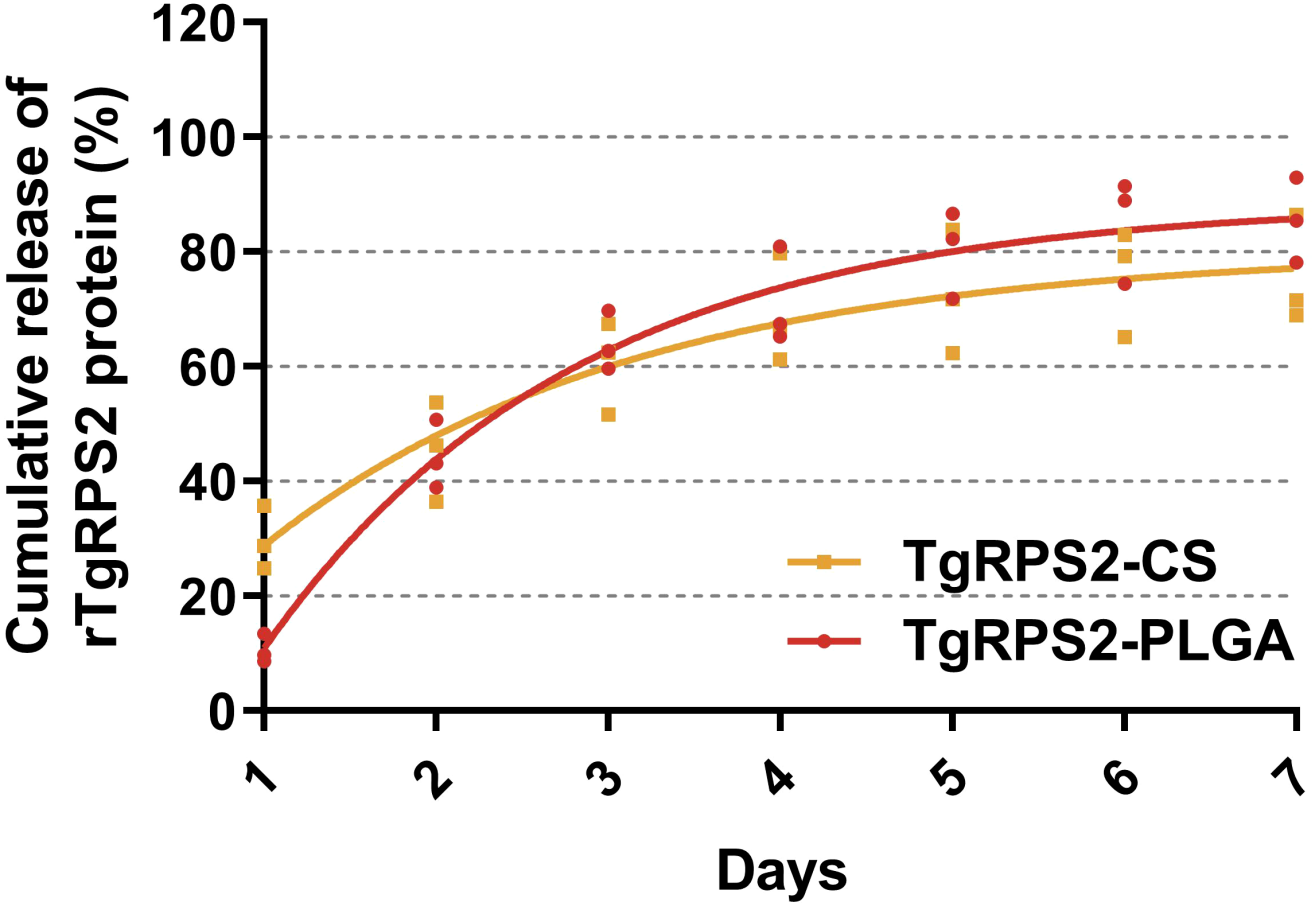
The release profile of PLGA and CS nanosphere loaded with TgRPS2 protein *in vitro* over 7 days. The released proteins were investigated by BCA methods and concentrations were evaluated by microplate reader. Each group had three replications and each replication was investigated once. Values are represented as mean ± SD (n = 3), and the dotted lines represent SD.

The release characteristics of the synthesized TgRPS2-PLGA and TgRPS2-CS nanospheres *in vitro* were evaluated by calculating different CR values at different points. As shown in **Figure 3**, two types of nanospheres exhibited a rapid release at the first three days, compared with the fourth to seventh day. The CR value of TgRPS2-CS nanospheres at the first detection was higher compared with TgRPS2-PLGA nanospheres, while the average CR value of TgRPS2-PLGA nanospheres was investigated higher during the third to seventh day. After the fourth day, the release curves of both nanospheres showed a slow and steady trend, continuing until the seventh day. The experimental results *in vitro* suggested the evidence that both obtained nanospheres could provide one-week-term stable release, which could enhance the effectiveness of immunizations.

### 3.5 Toxicity determination of synthesized nanospheres

**Figure 4:**
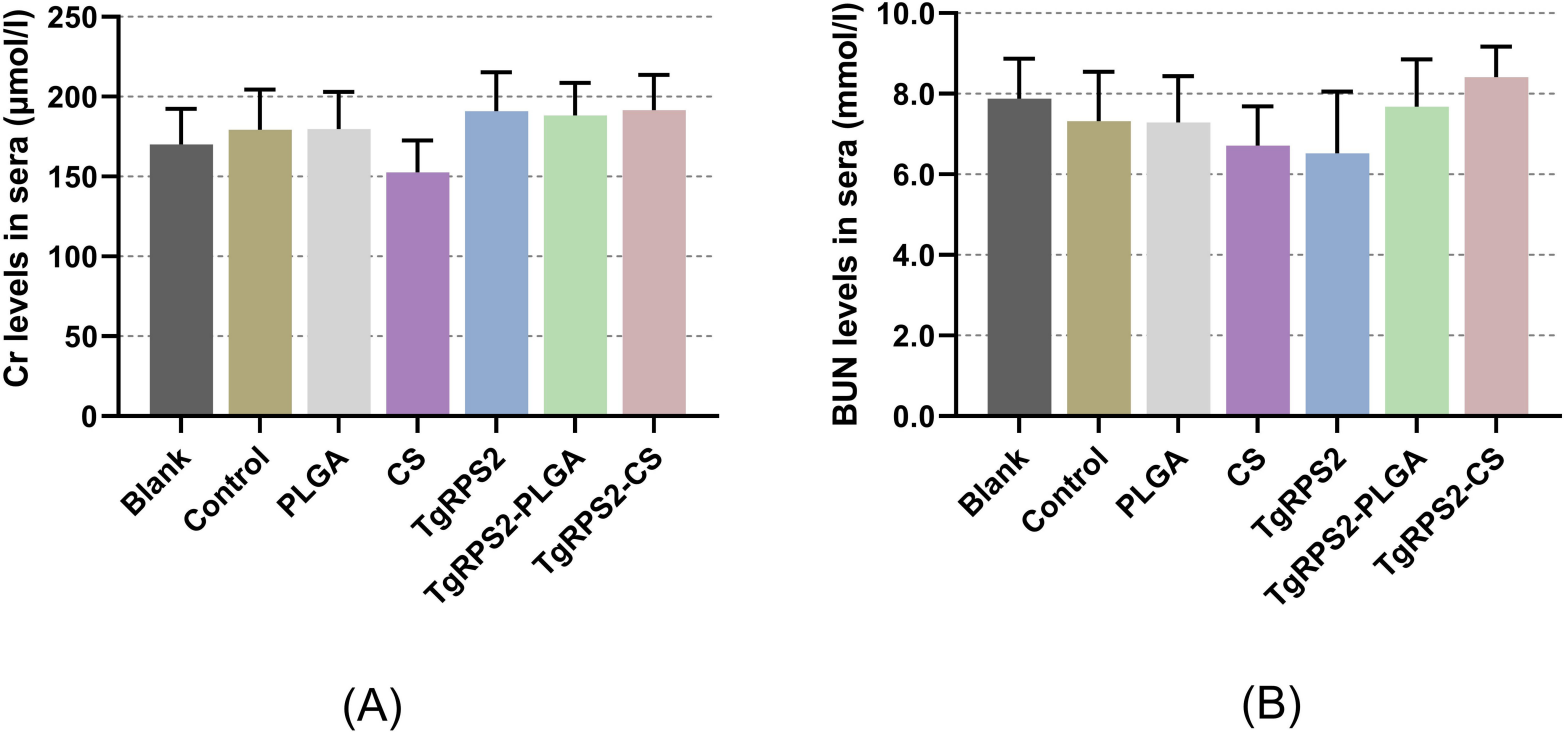
Toxicity analysis of TgRPS2 protein entrapped in PLGA and CS nanospheres. Sera were harvested from each group, and the levels of Cr **(A)** and BUN **(B)** were determined based on the instructions of commercial kits. Each serum was conducted once, and significance was evaluated by one-way ANOVA followed by Dunnett’s test. Values among the TgRPS2 protein, TgRPS2-PLGA, and TgRPS2-CS groups were pairwise compared by ANOVA following Bonferroni’s correction. Values are represented as mean ± SD (n = 5).

In order to investigate whether the nanospheres were poisonous, the levels of Cr and BUN in the collected sera were assessed based on the sarcosine oxidase assay and the urease-indole phenol assay. The results showed that the levels of Cr and BUN are within acceptable ranges, and no statistically significant difference was investigated among the immunization groups and the control group (*p* > 0.05, **Figure 4**). In addition, the behavior, diet, and physiological status of the animals were normal during the experiment, and no adverse reactions were observed. All these obtained results demonstrated that the synthesized nanospheres are non-toxic.

### 3.6 Evaluate antibodies and cytokines secretions in mice after vaccine immunization

**Figure 5:**
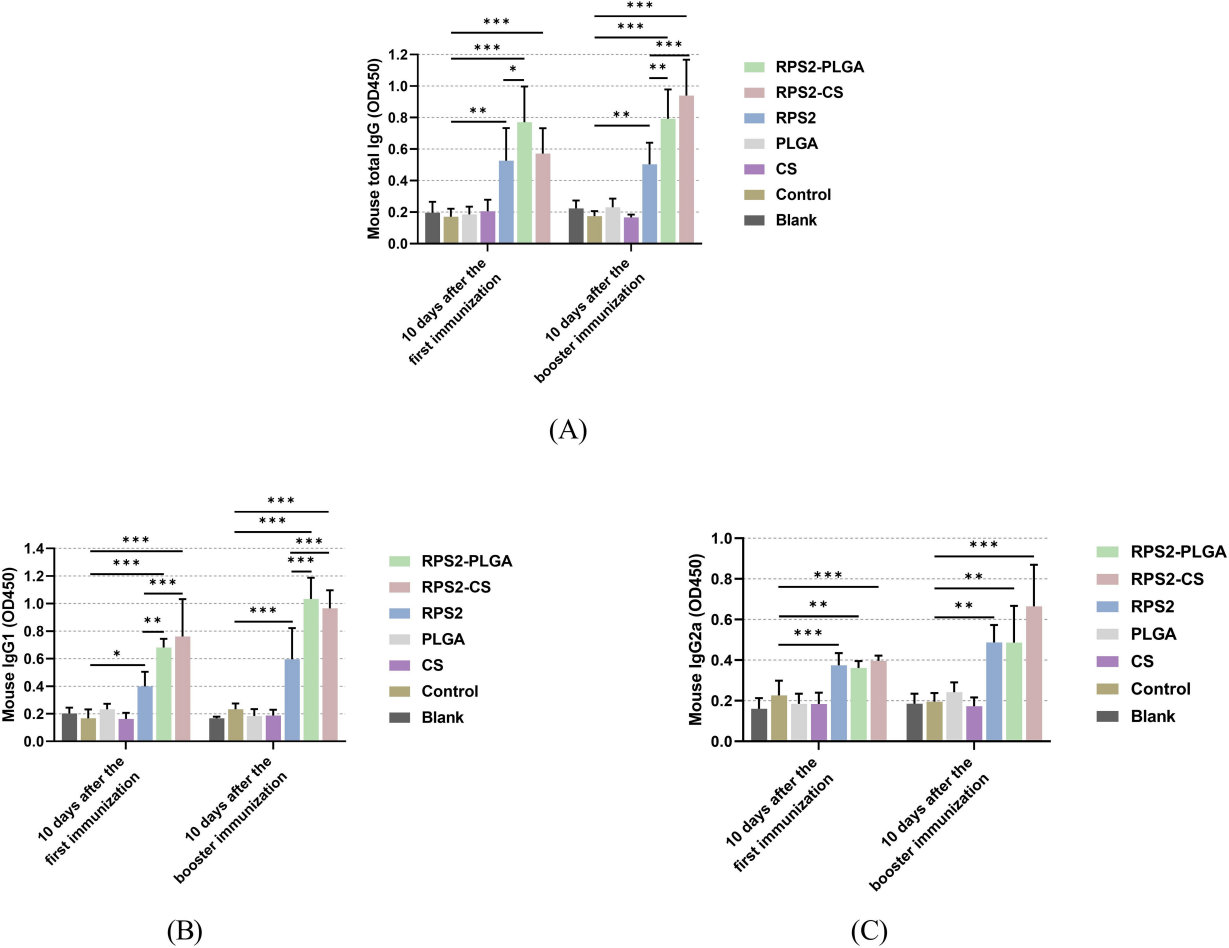
Secretions of total IgG **(A)**, isotypes IgG1 **(B)**, and IgG2 **(C)** in the sera from immunized mice at days 10 and 24. Each serum was conducted once, and significance was evaluated by one-way ANOVA followed by Dunnett’s test. Values among the TgRPS2 protein, TgRPS2-PLGA, and TgRPS2-CS groups were pairwise compared by ANOVA following Bonferroni’s correction. Values are shown as the mean of the OD450 ± SD (n = 5). *p < 0.05, **p < 0.01, and ***p < 0.001 compared with control group.

Based on the group assignment displayed in 2.9, sera were collected from all immunized mice to evaluate IgG, IgG2a, and IgG1 levels. As expected, vaccinations with two types of nanospheres and naked TgRPS2 protein could induce significantly higher levels of *T. gondii-*specific IgG when compared with the control group (*p* < 0.01, **Figure 5A**). Furthermore, TgRPS2-PLGA and TgRPS2-CS nanospheres could induce significantly higher levels of *T. gondii-*specific IgG when compared with naked TgRPS2 after booster immunization (*p* < 0.001). As shown in **Figures 5B, C**, mice immunized with recombinant proteins and its nanospheres generated higher levels of *T. gondii*-specific IgG1 and IgG2a when compared with the animals in the control group. It is noteworthy to note that regardless of the number of immunizations, immunization with TgRPS2-CS and TgRPS2-PLGA nanospheres could generate statistically enhanced IgG1 levels compared to the naked TgRPS2 protein (*p* < 0.01, **Figure 5B**).

**Figure 6:**
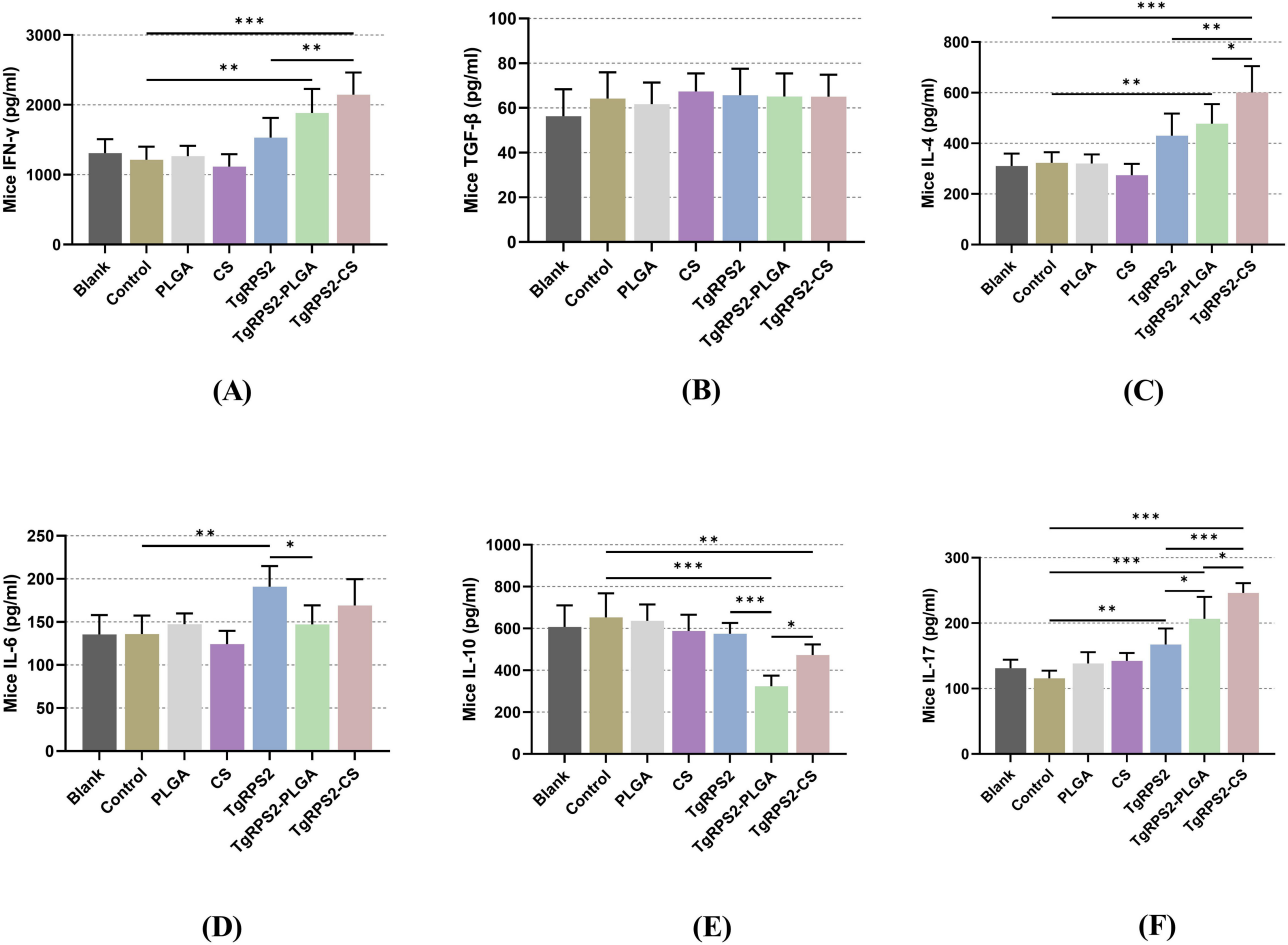
Cytokine-secretions of IFN-γ **(A)**, TGF-β **(B)**, IL-4 **(C)**, IL-6 **(D)**, IL-10 **(E)** and IL-17 **(F)** in animals’ sera after booster immunization. Commercially available ELISA kits were used to determine the level of cytokines in the sera from immunized mice. Each serum was conducted once, and significance was evaluated by one-way ANOVA followed by Dunnett’s test. Values among the TgRPS2 protein, TgRPS2-PLGA, and TgRPS2-CS groups were pairwise compared by ANOVA following Bonferroni’s correction. Values are shown as mean ± SD (n = 5). *p < 0.05, **p < 0.01, and ***p < 0.001 compared with control group.

After the booster immunization, the levels of IFN-γ, TGF-β, IL-4, IL-6, IL-10, and IL-17 in sera were measured by commercial ELISA kits to explore the effects of nanospheres on inflammation and immune response in the body. As demonstrated in **Figures 6A, C, F**, mice immunized with TgRPS2-PLGA and TgRPS2-CS nanospheres showed significantly increased secretions of IFN-γ, IL-4, and IL-17 compared with the controls (*p* < 0.01). The obtained results also showed that mice immunized with recombinant proteins generated significantly higher levels of IL-6 compared with the controls (*p* < 0.01, **Figure 6D**). In addition, the levels of IL-10 in the nanospheres-immunized groups, especially the TgRPS2-PLGA group, were significantly down-regulated compared to the control group (*p* < 0.001, **Figure 6E**). In addition, no statistically significant difference was detected in the levels of TGF-β between the control group and the immunized groups (*p* > 0.05, **Figure 6B**).

### 3.7 Phenotypic analysis of dendritic cells in immunized mice

**Figure 7:**
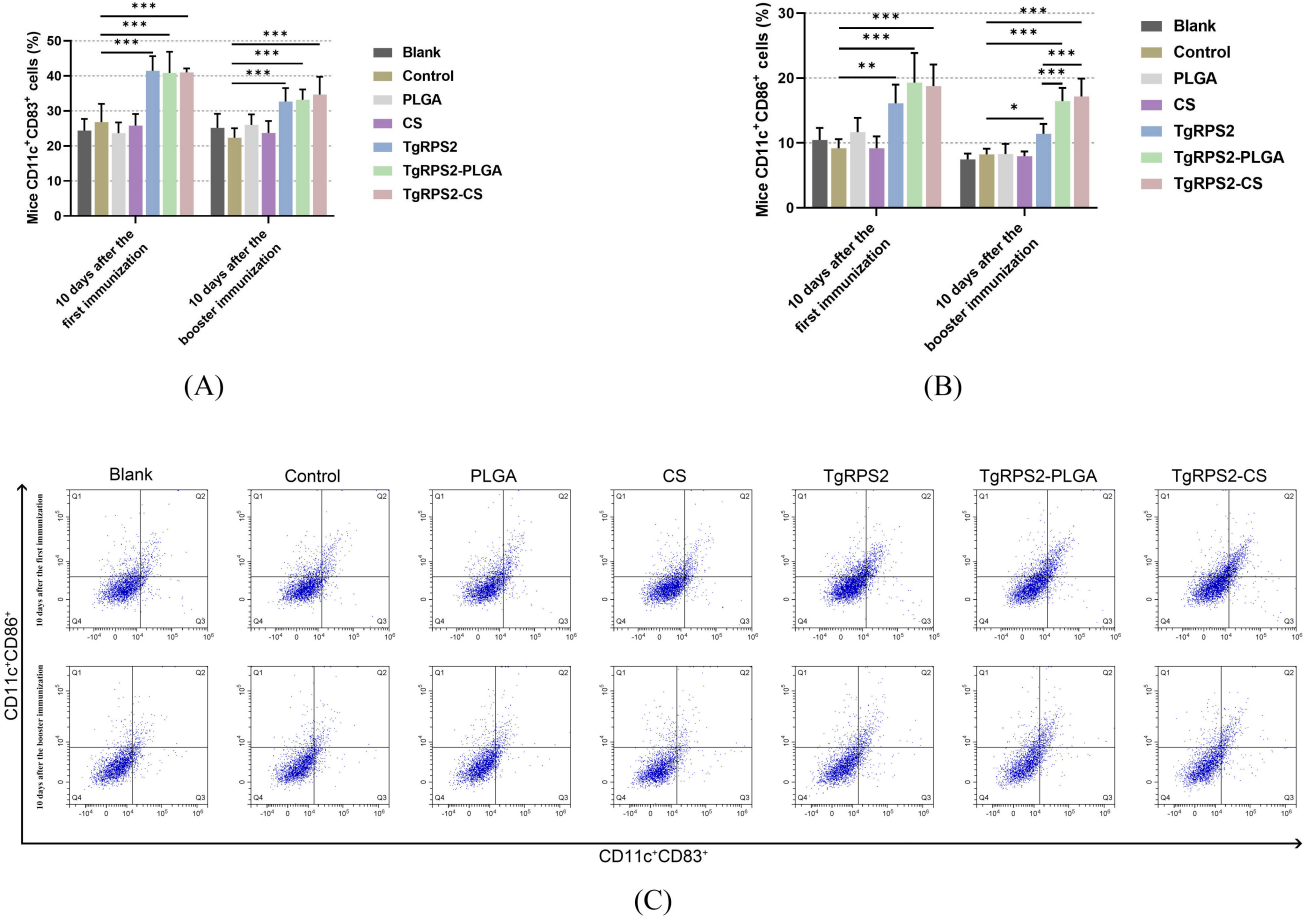
Flow cytometry analysis of CD83^+^ **(A)** and CD86^+^ molecules **(B)** on splenic DCs collected from immunized mice. Five mice in each group were sacrificed, and spleen lymphocytes from each mouse were investigated. The dot plots **(C)** showed the relative number of CD11c^+^CD83^+^ and CD11c^+^CD86^+^ cells. Values are shown as mean ± SD (n = 5), and significance was evaluated by one-way ANOVA followed by Dunnett’s test. Values among the TgRPS2 protein, TgRPS2-PLGA, and TgRPS2-CS groups were pairwise compared by ANOVA following Bonferroni’s correction. *p < 0.05, **p < 0.01, and ***p < 0.001 compared with control group.

Dendritic cells (DCs) subpopulations in the collected spleen lymphocytes were determined to evaluate the maturation of DCs by flow cytometry. Compared with the controls, TgRPS2 protein, TgRPS2-PLGA, and TgRPS2-CS nanospheres could significantly induce the expression levels of CD83 and CD86 in lymphocytes (*p* < 0.001, **Figures 7A–C**). Moreover, the expressions of CD86 in the TgRPS2-PLGA and TgRPS2-CS groups were significantly higher than that in the TgRPS2 group after the booster vaccination (*p* < 0.001, **Figure 7B**). All these results suggested that synthetic nanospheres and TgRPS2 proteins play an important role in stimulating lymphocytes to generate CD83 and CD86 molecules.

**Figure 8:**
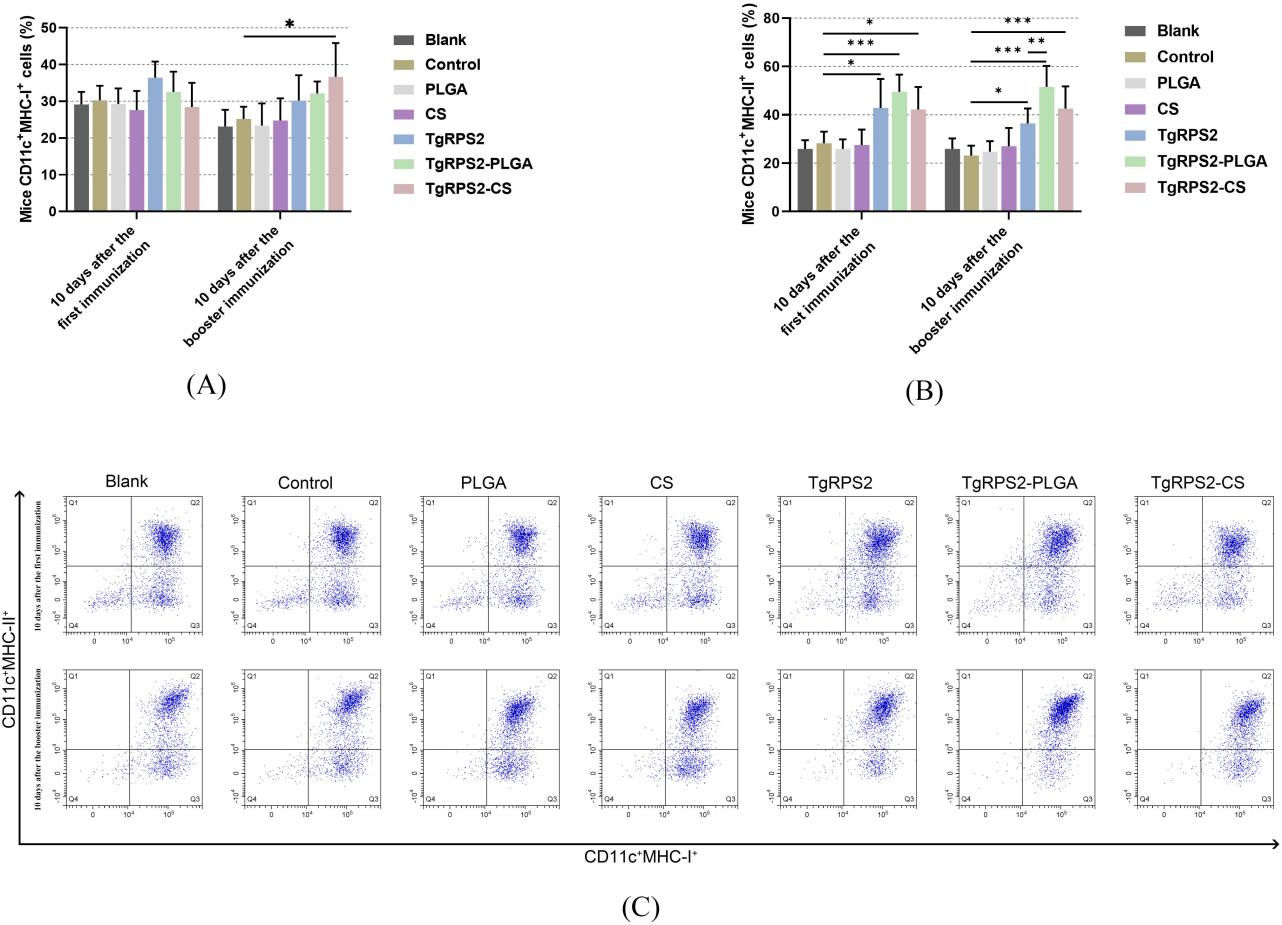
Flow cytometry analysis of MHC-I **(A)** and MHC-II molecules **(B)** on the surface of DCs in immunized mice. Five mice in each group were sacrificed, and spleen lymphocytes from each animal were investigated. The dot plots **(C)** showed the relative number of CD11c^+^MHC-I^+^ and CD11c^+^MHC-II^+^ cells. Values are shown as mean ± SD (n = 5), and significance was evaluated by one-way ANOVA followed by Dunnett’s test. Values among the TgRPS2 protein, TgRPS2-PLGA, and TgRPS2-CS groups were pairwise compared by ANOVA following Bonferroni’s correction. *p < 0.05, **p < 0.01, and ***p < 0.001 compared with control group.

To investigate the effects of PLGA and CS nanospheres on antigen presentation, the expression of MHC-I and MHC-II in all immunized groups was analyzed by flow cytometry. As shown in **Figures 8A, C** the expression levels of MHC-I in DCs collected from the TgRPS2-CS group were detected significantly higher after the enhanced immunization when compared with the control group (*p* < 0.05). Compared with the controls, TgRPS2 protein, TgRPS2-PLGA, and TgRPS2-CS nanospheres could significantly promote the expression levels of MHC-II in DCs (*p* < 0.05, **Figures 8B, C**). Furthermore, the expressions of MHC-II in TgRPS2-PLGA groups were significantly higher than that in the TgRPS2 group after the booster vaccination (*p* < 0.01, **Figure 8B**). These results suggested that the TgRPS2 protein and its nanospheres, especially PLGA nanospheres, could activate the antigen-presenting effect of DCs.

**Figure 9:**
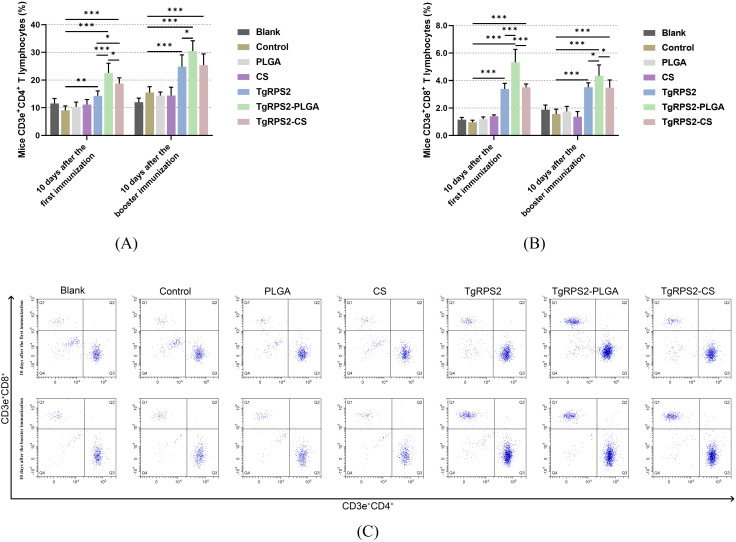
Flow cytometry analysis of CD4^+^ **(A)** and CD8^+^ T lymphocytes **(B)** in splenic lymphocytes harvested from immunized mice. Five mice in each group were sacrificed, and spleen lymphocytes from each animal were investigated. The dot plots **(C)** showed the relative proportion of CD3e^+^CD4^+^ and CD3e^+^CD8^+^ cells. Values are shown as mean ± SD (n = 5), and significance was evaluated by one-way ANOVA followed by Dunnett’s test. Values among the TgRPS2 protein, TgRPS2-PLGA, and TgRPS2-CS groups were pairwise compared by ANOVA following Bonferroni’s correction. *p < 0.05, **p < 0.01, and ***p < 0.001 compared with control group.

CD4^+^ and CD8^+^ T lymphocytes played a pivotal role in the control of *T. gondii* infections. To investigate the inductions of PLGA and CS nanospheres on T lymphocyte response, the expression levels of CD4^+^ and CD8^+^ T lymphocytes in the collected spleen lymphocytes were evaluated by flow cytometry. As shown in **Figures 9A–C**, compared with the controls, TgRPS2 protein, TgRPS2-PLGA, and TgRPS2-CS nanospheres could significantly increase the expression levels of CD4^+^ and CD8^+^ T lymphocytes in lymphocytes (*p* < 0.05). After the initial immunizations, the expression levels of CD4^+^ and CD8^+^ T lymphocytes in the nanospheres-immunized groups, especially the TgRPS2-PLGA group, were significantly higher compared to the TgRPS2 protein-immunized group *(p* < 0.05, **Figures 9A, B**). These obtained findings demonstrated that the TgRPS2 protein, as well as its two types of nanospheres, could stimulate lymphocytes to generate CD4 and CD8 molecules, and PLGA nanospheres exhibited a great advantage in activating T lymphocytes.

### 3.8 Lymphocyte proliferation was assessed in immunized mice

**Figure 10:**
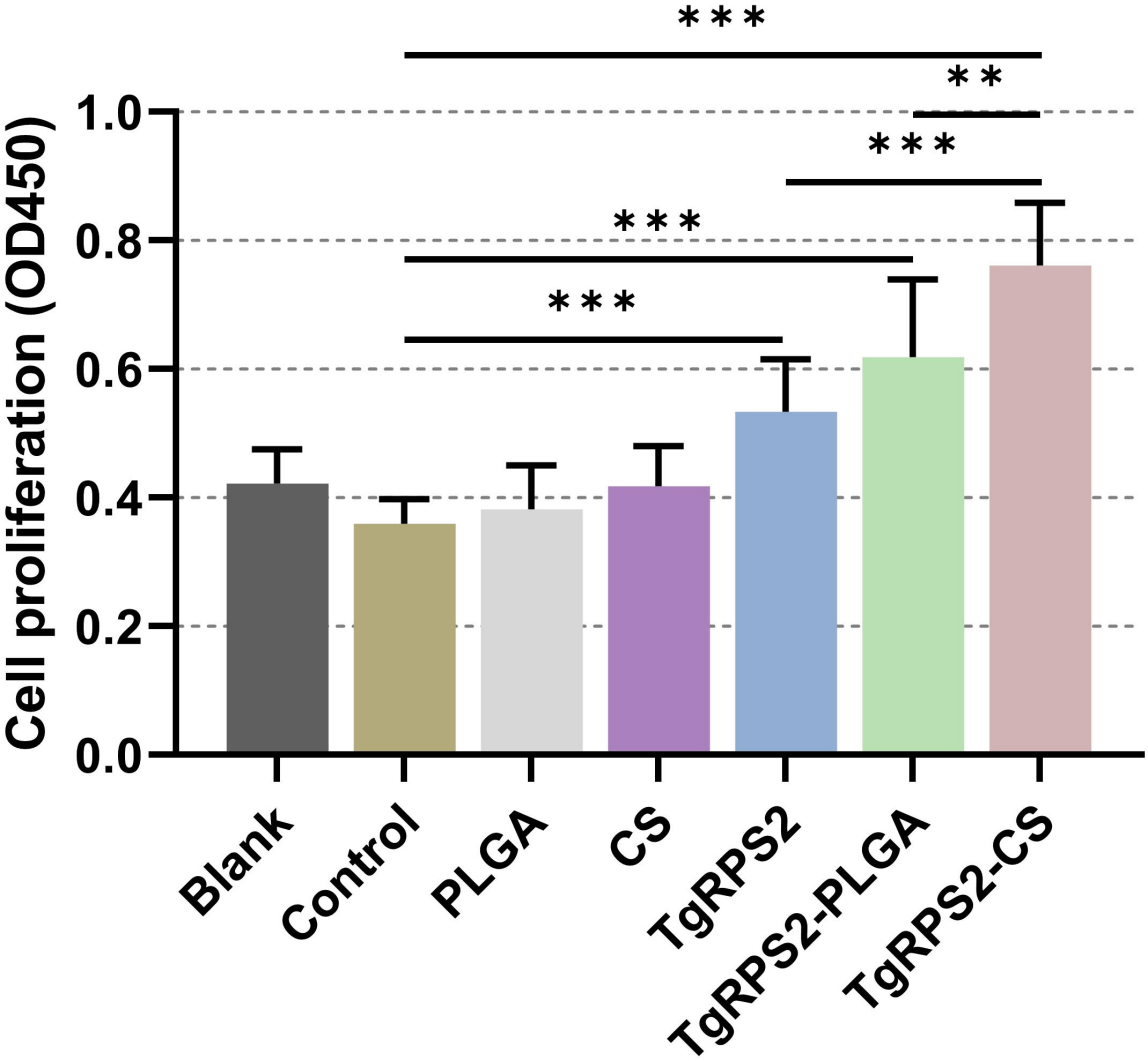
Splenocyte proliferation of immunized mice. Three mice in each group were sacrificed and the lymphocytes from each mouse were collected, the obtained lymphocytes were then divided into three parts, and each part of lymphocyte were cultured with 10 μg/ml rTgRPS2 protein. Each part of lymphocytes was investigated once, and significance was estimated by one-way ANOVA followed by Dunnett’s test. Values are shown as mean ± SD (n = 9), and significance was evaluated by one-way ANOVA followed by Dunnett’s test. Values among the TgRPS2 protein, TgRPS2-PLGA, and TgRPS2-CS groups were pairwise compared by ANOVA following Bonferroni’s correction. **p < 0.01 and ***p < 0.001 compared with control group.

The day before the challenge experiment (on the 28th day), spleen lymphocytes were isolated from the immunized mice, and the status of lymphocyte proliferation was assessed using commercial CCK-8 kits. As shown in **Figure 10**, lymphocyte proliferation was significantly increased in the nanospheres-immunized group compared with the control group (*p* < 0.001). In addition, compared with the TgRPS2 protein, TgRPS2-CS nanospheres could statistically induce the proliferation of lymphocytes (*p* < 0.001). These observations were indicative of a great effect of TgRPS2 protein, and its two types of nanospheres, in stimulating T-lymphocyte proliferation.

### 3.9 Immunohistochemical analysis of the spleen from immunized mice

**Figure 11:**
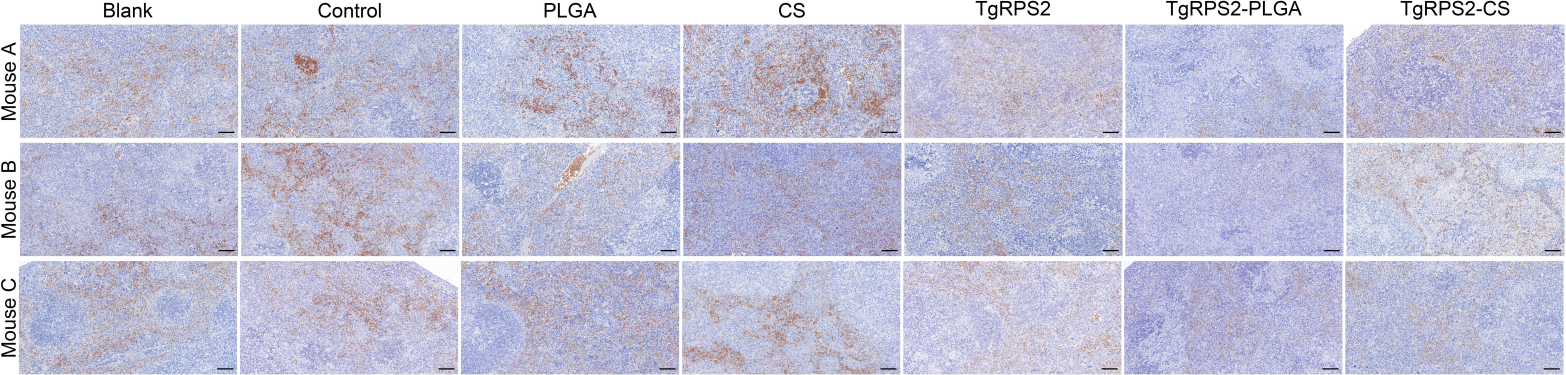
Immunohistochemistry section observation of spleens collected from challenged mice. Four days after the challenge, three animals were euthanized and the spleen tissues were harvested, each spleen was made into one paraffin section (n =3). One horizon of each section was randomly selected at the high magnification (bar represented 100 μm).

After 14 days of the booster immunization (on the 29th day), 100 highly virulent *T. gondii* RH strain tachyzoites were intraperitoneally injected into mice for challenge experiments. Four days later (On the 33 days), the mice were sacrificed under the supervision of the Ningxia University Technology Ethics Committee, and spleen tissues were collected and processed into paraffin sections. As demonstrated in **Figure 11**, mice immunized with TgRPS2 protein, TgRPS2-PLGA, and TgRPS2-CS nanospheres exhibit reduced spleen infection by *T. gondii.* These findings suggested that nanospheres-immunization could provide immune protection in mice against toxoplasmosis, and such resistance could inhibit the growth of *T. gondii* tachyzoites to some extent.

### 3.10 *T. gondii*-burdens in cardiac tissues

**Figure 12:**
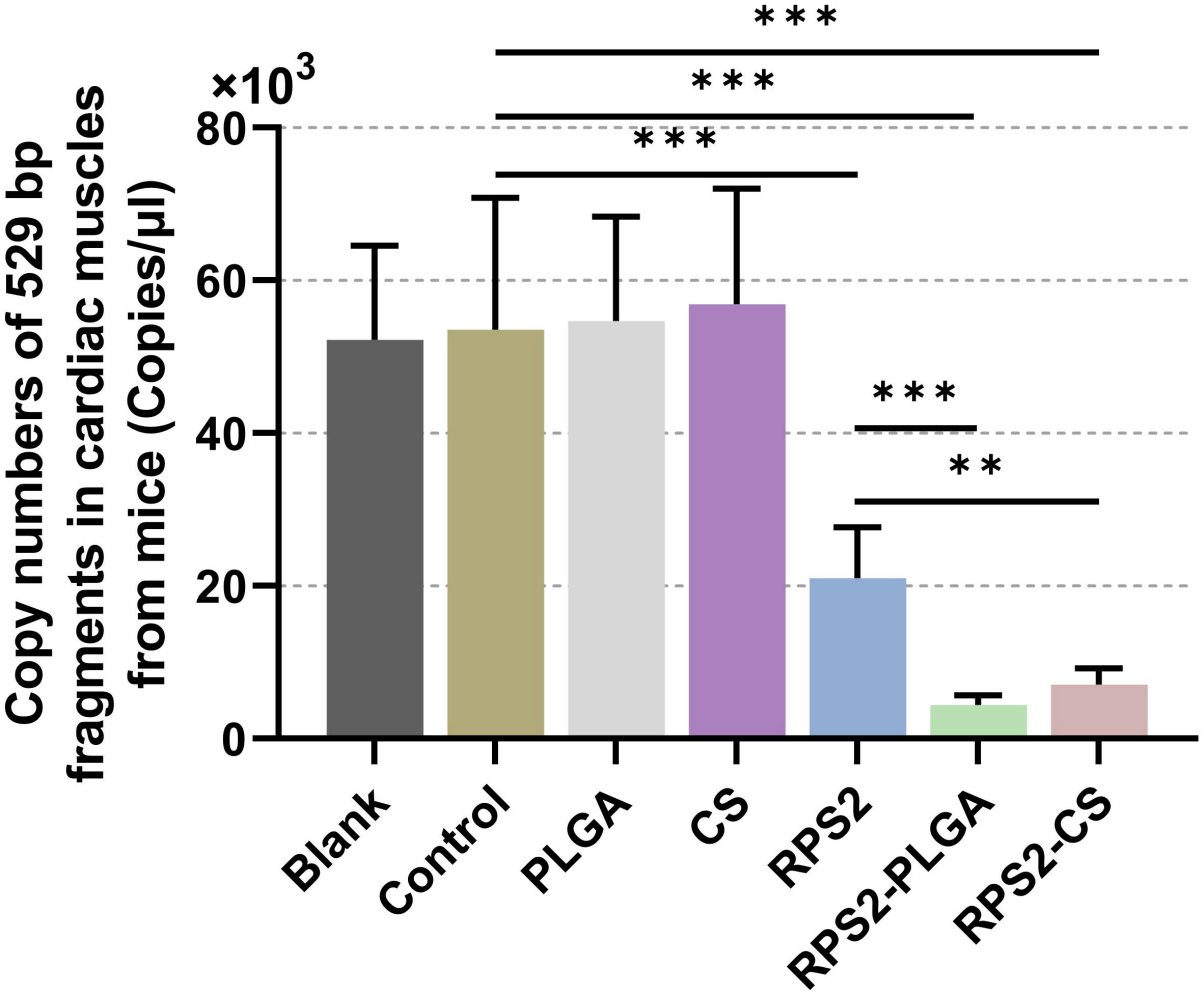
Copy numbers of *T. gondii* 529 bp fragments in cardiac muscles. Immunized mice were challenged with 100 tachyzoites two weeks after the booster immunization. Six days after the challenge, ten cardiac muscles were harvested and the parasite burdens were investigated by qPCR. Each sample was investigated three times (n = 15) and values were analyzed by one-way ANOVA analysis followed by Dunnett’s test. Values between the TgRPS2 protein, TgRPS2-PLGA, and TgRPS2-CS groups were estimated by the independent t-test. Values were shown as mean ± SD (n = 15). **p < 0.01, ***p < 0.001 compared with control group.

After 14 days of the booster immunization (on the 29th day), all groups of mice were challenged with 100 highly virulent *T. gondii* RH strains. Then, six days after the challenge (On the 35th day), qPCR was conducted to evaluate the parasite burdens in heart tissues harvested from immunized mice. Compared to the control group, the parasite burdens in TgRPS2, TgRPS2-PLGA, and TgRPS2-CS nanospheres groups were significantly reduced by 60.7%, 91.7%, and 86.7%, respectively. In addition, the parasite burdens in the TgRPS2-PLGA and TgRPS2-CS groups were statistically reduced compared with the TgRPS2 group (*p* < 0.01, **Figure 12**). The obtained results demonstrated that the immune response generated by synthesized nanospheres played an essential role in controlling *T. gondii* infection.

## 4 Discussion

Toxoplasmosis generates significant damage to public health, emphasizing the need to develop a correct way against *T. gondii.* Despite the continuous investigation of various vaccines for *T. gondii*, no suitable vaccine has been found to provide complete protection for *T. gondii* infection so far (32). Therefore, it is urgent to develop a safe and effective vaccine against *T. gondii*, and the emergence of nano vaccines has brought hope for this. Currently, nano vaccines outnumber conventional vaccines due to their plasticity in physical and chemical properties and ease of administration (33). Nanospheres can maintain the natural conformation of antigens at the site of the immune attack and gradually release antigens locally over time, thereby producing a depot effect, which is a key advantage of these types of nanospheres (34). Research has shown that nanospheres have possessed great potential in resisting *T. gondii* tachyzoites and bradyzoites (35). Furthermore, Nanospheres have a certain degree of immunogenicity, making it have become an attractive and strong candidate for infectious diseases, such as hepatitis B and toxoplasmosis (36, 37).

Natural polymeric nanospheres are popular as delivery vehicles in prophylactic vaccine formulations due to their unique properties (37). Previous research has shown that the vaccine delivery by PLGA generated better immune protection than the traditional alum adjuvant (38). In addition, chitosan as a presentation carrier could promote Th1 cell immune responses (39), and the absorbed chitosan also could induce mitochondrial stress to trigger the production of reactive oxygen species, which are key responses to resist *T. gondii* (39, 40). Approved by the FDA, PLGA and chitosan have been used as vaccines and drug delivery (41, 42). In addition, appropriate antigen selection is equally important as the delivery system. As the apicoplast genome encoded proteins with a signal peptide, *T. gondii* ribosomal protein S2 can be carried to the cytoplasm or membrane of *T. gondii* (43, 44). According to the previous studies on antigenic potential analysis, *T. gondii* RPS2 could serve as a potential antigen, due to its hypo-allergenic and soluble characteristics (44). Studies have found that rTgRPS2 can be recognized by the natural ESA of *T. gondii* tachyzoites, located at the top of the tachyzoites, and may participate in the invasion or exit of *T. gondii* tachyzoites (16). Recombinant proteins have good safety and do not pose a risk of reinfection. Choosing nanospheres as the delivery system for vaccines can protect proteins from adverse degradation and help them to be effectively absorbed by host cells. In the current study, we explored the possibility of utilizing the *T. gondii* ribosomal S2 protein as a vaccine candidate to prevent acute toxoplasmosis in animals, and the nano vaccines were synthesized by using two types of nanospheres. Freeze-dry the synthesized nanospheres for long-term storage at 4°C.

Nano vaccines gained great advantages in enhancing the immunoprotections in resisting pathogens (45, 46). Nanospheres with a diameter of 20-200 nm can freely flow into lymph node (LN) resident DC or macrophages (47), and the size of nanospheres ranging from 20 to 200 nm may enhance the uptake and presentation of the antigens by Antigen-presenting cells (APC) (48). The PLGA nanospheres with a diameter of less than 500 nm can elicit more effective CTL reactions (49). Moreover, nanospheres with an average diameter of 200 nm could enhance the proportion of antigen-specific polyfunctional CD4 T cells compared to 30 nm (50). These studies showed that the particle size of nanospheres has a certain influence on its immunogenicity (51). In recent research on nano vaccines, the average diameter of nanospheres was about 40-500nm and exhibited good immune effect (52, 53). In our research, the synthesized nanospheres, observed by SEM, were spherical with uneven surface textures, and the diameter of the nanospheres was approximately 100nm. These results showed that the nanospheres we have synthesized could generate good immune effects.

An increasing number of studies suggested that nanospheres possess a sustained-release capability (54, 55). According to the investigation of the nanospheres release effect, previous studies showed that PLGA nanospheres elicited prolonged antibody titers (56), and continuous antigen delivery mediated by nanospheres is crucial for the long-term quality and intensity of vaccine response. Previous studies showed that encapsulation proteins and peptides into PLGA nanospheres could hydrolyze in aqueous media for approximately 20–30 days, which could be the key to triggering a strong and sustained cytotoxic T-cell response (57). Chitosan-coated alginate exhibits a relatively slow and sustained release of pIL-1β, with studies demonstrating that the release experiment can last up to 28 days (58). In our study, two types of nanospheres, TgRPS2-PLGA and TgRPS2-CS showed good sustained-release performance and complete release generally took about 7 days. This could be related to the type of antigen presented, nanospheres size, porosity, and molecular weight size (59). In addition, TgRPS2-CS nanospheres showed a slightly lower release profile than TgRPS2-PLGA, possibly because CS is a negatively charged polymer that can bind to cationic polymers on the cell surface and stay briefly in target cells to slowly release antigens (60). These results demonstrated that the nanospheres we have developed can effectively improve the immunogenicity of vaccine antigens.

Toxicity evaluation is a necessary step before clinical testing of vaccines. The BUN and Cr are valuable screening tests in evaluating renal disease (61). Therefore, we determined the toxicity of the vaccine by administering excessive doses to mice. In this study, we fully freeze-dried TgRPS2-PLGA and TgRPS2-CS nanospheres during vaccine preparation and sealed them at 4°C to facilitate the removal of DCM and long-term storage. By toxicity determination of synthesized nanospheres, BUN and CR were maintained within normal levels and the behavior, diet, and physiological status of the animals were normal during the experiment, demonstrating that the synthetic nanospheres were non-toxic.

The generation of humoral immune memory against pathogens is critical for a successful vaccine, and the immune response mediated by circulating memory B cells and neutralizing antibodies provides a strong defense against infection (62, 63). As previously reported, IgG1 antibody subtype was associated with Th2-related immunity, while the Th1-related immunity was related to IgG2a subtype (64). In our current research, the obtained data showed that mice immunized with nanospheres could produce higher levels of IgG as well as its subtypes (IgG1 and IgG2a), emphasizing that nanospheres could induce mixed Th1/2 immune responses, and such observations are consistent with previous reported studies (65, 66). In addition, the mice immunized with two types of nanospheres produced higher levels of IgG, IgG1, and IgG2a, compared with the animals immunized with TgRPS2 protein alone, such results indicated the synthesized nano vaccines could induce stronger humoral immunity than the naked antigens. As previously reported, Nabi et al. found that *T. gondii* ROP18 encapsulated in PLGA nanospheres could significantly increase the secretion of IgG2a (67). Similarly to our results, Xu et al. also reported the promotion effects of IgG1 and IgG2a in mice immunized with PLG-rROP38-rROP18 nanospheres (68). In summary, our results demonstrated that TgRPS2-PLGA and TgRPS2-CS nanospheres could enhance mice’s immune efficacy against *T. gondii* to some extent.

The strong innate immune response triggered by macrophages (MΦ), DCs, and neutrophils (Np) is crucial for early *Toxoplasma* infection (69, 70). Toll-like receptors (TLR), IFN-γ induced GTPases, and inflammasomes are three major branches of innate immune sensing (71), among which IFN-γ driven immune responses are essential for against *T. gondii* infections (72). Meantime, IL4 also has a great advantage against infection, as it can enhance the production of INF-γ to reduce the tissue cysts caused by *T. gondii* (73). In this research, IFN-γ and IL-4 in nano vaccine groups were significantly increased in animals after the immunization. This demonstrated that two types of nanospheres trigger IFN-γ immune responses. Although immune responses are crucial for resisting pathogens, excessive responses can cause severe damage to the body and even lead to death (74). Therefore, a negative regulator of inflammatory responses is equally important for maintaining the balance of the body against parasites. IL-10 produced by Th1 cells can limit excessive inflammatory responses and avoid immunopathological responses (74). Another critical cytokine involved in regulating immunopathology modulation is TGF-β, and many reported studies suggested that TGF-β alone may be insufficient to generate efficacy immunoprotection during *T. gondii* infection and requires additional down-regulation activity of IL-10 (75). In our research, compared with the controls, mice immunized with TgRPS2-PLGA and TgRPS2-CS nanospheres showed down-regulated IL-10 levels. Furthermore, no statistically significant difference was observed in TGF-β levels between nanospheres and the control group. Such phenomena demonstrated that the possibility of nanospheres could regulate the expression of IL-10 during vaccine immunizations. As reported, IL-6 participates in the early development of acute phase response in mice with *T. gondii* encephalitis (76). In the current study, the increase of IL-6 levels after immunization with TgRPS2 protein suggests that the antigen may be involved in the inflammatory response. In addition, there was no significant difference in IL-6 levels between the nanosphere immunization group and the control group, which may be related to factors such as vaccine type and individual differences in the host. Th17 immunity is also involved in resisting parasites as previously reported, and Th17 cells could secrete cytokine IL-17 and promote inflammatory response during parasitic infections (77, 78). Meantime, IL-17 also shows significant protective effects in *T. gondii* infection and IL-17-deficient mice are more susceptible to acute infection with the parasite (79). In our research, the levels of IL-17 were elevated in immunized mice, suggesting that the nanospheres also mobilized Th17 cells to participate in the anti-parasitic response. All these results demonstrated that the synthesized nanospheres could regulate cytokines in animals, trigger Th1/17 cell responses, and mobilize the body’s immune response.

Belong to the antigen-presenting cells, DCs mediate the transition between innate and adaptive immune responses, and its activation and maturation are crucial in controlling *T. gondii*-infection (80). As the most characteristic cell surface marker of fully mature DCs in the peripheral circulation, the CD83 molecule modulates the immune response by regulating the maturation of T and B-lymphocytes (81–84). As a co-stimulatory molecule, CD86 can effectively activate T cells to induce adaptive immunity and play an important role in regulating antigen presentation in DCs (85). Thus, in this study, we investigated the proportion of CD83 and CD86 through flow cytometry. The results showed that CD83 and CD86 molecules were significantly increased in all nanosphere-immunized mice, suggesting that TgRPS2-PLGA and TgRPS2-CS nanospheres could contribute to the activation and maturation of DCs. Mature DCs could generate MHC-I and MHC-II molecules to conduct antigen presentation (86). MHC-II molecules are ligands for CD4 T cells, while the molecular weight of MHC-I is related to the potential activity of anti-*T. gondii* lysate CD8 T cells (87). According to the observations of the present study, the levels of MHC-II molecules were remarkably increased in animals immunized with both types of nanospheres, whereas the levels of MHC-I were upregulated only in animals immunized with the TgRPS2-CS nanosphere. All the obtained results indicated that TgRPS2-PLGA and TgRPS2-CS nanospheres could promote antigen presentations in DCs, and such results were consistent with TG290 mRNA-LNP, reported by Li et al. (88).

T-cell activation is essential for resistance to *T. gondii* infection (89). Mature DCs could migrate into secondary lymphoid tissue and interact with immature antigen-specific T cells (90, 91), causing T cells to differentiate into CD4 and CD8 T cells. Previous studies revealed that patients lacking CD4 and CD8 T cells are susceptible to toxoplasmosis and predisposed to toxoplasmas encephalitis in chronic infections (92, 93). CD4 T cells play a central role in immune protection, mainly coordinating the entire immune response by producing cytokines and chemokines (94). It is essential for regulating the functional activity of CD8 T cells in the brain (95). Activated CD8 T cells can generate cytotoxic T lymphocytes (CTLs) and then exhibit immunotoxicity to parasites (96). According to our study, nanospheres-immunized mice generated higher levels of CD4 and CD8 T lymphocytes than controls, suggesting that TgRPS2 plays an essential role in inducing T-lymphocytes against *T. gondii*. As the key indicator, the proliferation of lymphocytes is the most important index reflecting organic immunity *in vivo* (97). In this study, the synthesized nanospheres could significantly promote lymphocyte proliferation, demonstrating nanospheres can induce T-cell activation, promote lymphocyte proliferation, and enhance immune protection. Report by Sun et al. (100), GRA7-pEGFP-C2^+^ nanospheres could enhance the immune response against the infections of *T. gondii*. Similar findings were also found in mice vaccinated with chitosan nanospheres loaded with CS-SAG1-PRO antigens (101).

The challenge experiments are the key standard to evaluate the vaccines. Therefore, mice were challenged with 100 *T. gondii* virulent RH strain. Based on previous research, the immunoprotective effect of synthesized nanospheres was evaluated by calculating *T. gondii*-burdens in heart tissue (97). In our study, mice immunized with TgRPS2-PLGA and TgRPS2-CS nanospheres, especially the TgRPS2-PLGA nanospheres, showed lower cardiac burden compared to naked TgRPS2 protein. By conducting quantitative competitive PCR conducted by the previous study, *T. gondii* first colonizes Peyer’s patches, followed by ileal parenchyma, mesenteric lymph nodes, spleen, portal vein, and aortic blood after peroral infection. In addition, the unique morphology and functional differences of the spleen are more conducive to expressing the dynamic complexity of infection (98, 99). Therefore, four days after the challenge, the spleen of mice was collected and paraffin sections were made to evaluate the immunoprotective effect. The results of the spleen section showed a significant reduction in tissue invasion by TgRPS2-PLGA and TgRPS2-CS nanospheres. The results of paraffin sections are consistent with those in myocardial tissue. In conclusion, TgRPS2-PLGA and TgRPS2-CS nanospheres could be a promising candidate vaccine against *T. gondii* infection.

In this study, TgRPS2-PLGA and TgRPS2-CS were synthesized as nano vaccines by encapsulating rTgRPS2 in PLGA and chitosan, and their protective efficacy was evaluated by immunizing ICR mice according to the immunization table. The research results suggested that the ability of TgRPS2-PLGA and TgRPS2-CS nanospheres to induce *T. gondii*-specific antibodies and cytokine production is similar. TgRPS2-CS has more advantages than TgRPS2-PLGA nanospheres in promoting lymphocyte proliferation. However, the cardiac load of mice immunized with TgRPS2-PLGA nanospheres was lower than that of TgRPS2-CS nanospheres. Therefore, we think that TgRPS2-PLGA and TgRPS2-CS nanospheres could induce satisfactory immune protection, but TgRPS2-PLGA nanospheres are slightly superior to TgRPS2-CS nanospheres in resisting acute toxoplasmosis.

## 5 Conclusion

In the current study, we investigated the feasibility of *T. gondii* ribosomal protein S2 delivered by PLGA and CS nanospheres as the nano vaccines against acute toxoplasmosis. The results suggested that the two synthesized nanospheres can enhance the immunoprotective effect of TgRPS2. They could activate innate immunity, enhance cellular immunity, and trigger Th1/2 immune responses by regulating the levels of antibodies and cytokines, providing immune protection against acute *T. gondii* infection. In addition, TgRPS2-PLGA and TgRPS2-CS nanospheres, as known as the nano vaccines, can significantly inhibit the invasion of splenic parasites and reduce parasite load in animals. Therefore, TgRPS2-PLGA and TgRPS2-CS nanospheres could be the appropriate candidates for preventing acute toxoplasmosis. However, chronic toxoplasmosis poses a significant threat to animals, so we need to validate its role in the chronic stage phase of *T. gondii* infection in the future.

## Data Availability

The original contributions presented in the study are included in the article/supplementary material. Further inquiries can be directed to the corresponding author/s.
